# The Burden of Poor Reproductive Health in England: Results From a Cross‐Sectional Survey

**DOI:** 10.1111/1471-0528.18133

**Published:** 2025-03-24

**Authors:** Melissa J. Palmer, Ona L. McCarthy, Rebecca S. French

**Affiliations:** ^1^ Department of Public Health, Environments, and Society, Faculty of Public Health and Policy London School of Hygiene and Tropical Medicine London UK

**Keywords:** England, inequalities, reproductive health

## Abstract

**Objective:**

To quantify the burden of poor reproductive health in England by age, ethnicity, and financial security.

**Design:**

Cross‐sectional survey.

**Setting:**

England.

**Sample:**

59 332 women and people assigned female at birth aged 16–55 years.

**Methods:**

The Reproductive Health Survey for England 2023 (RHSE2023) used an online convenience sampling strategy and a self‐completion questionnaire.

**Main Outcome Measures:**

13 indicators of reproductive health organised into three domains: reproductive morbidities (including endometriosis, fibroids); menstrual health (severely painful and/or heavy periods; menopausal symptoms); and pregnancy‐related adverse experiences (pregnancy loss, infertility, unplanned pregnancy) in the last year.

**Results:**

Compared to the general population, our sample over‐represented those with higher education levels and under‐represented minority ethnic groups. 28.0% of participants reported at least one reproductive morbidity; 61.9% reported menstrual‐related issue(s); and 5.5% reported pregnancy‐related adverse experience(s) in the last year, with considerable variation by age. Compiling the three domains, 73.7% reported at least one indicator of poor reproductive health. Inequalities were observed: Black British, Caribbean, and African women had increased odds of reporting reproductive morbidity (aOR: 1.69); heavy and/or severely painful periods (aOR: 1.28); and pregnancy‐related adverse experience (aOR: 1.50). Financial insecurity was also associated with poor reproductive health.

**Conclusions:**

As the first study to simultaneously examine this broad range of indicators of reproductive health within a single sample, we highlight the substantial burden of poor reproductive health in England, with evident ethnic and financial inequalities.

## 1 Introduction

Reproductive health is central to overall health and wellbeing. A multitude of conditions and experiences can impact a person's reproductive health, and needs and priorities change according to age and life‐stage. Understanding the range and prevalence of reproductive health issues across the life course is crucial so that efforts to improve reproductive health can be appropriately targeted.

Inequalities in reproductive health are well documented; for example, deprivation has been shown to be associated with higher sexually transmitted infection and abortion rates [1], risk of severe maternal morbidity [2] and adverse pregnancy outcomes [3]. There is also considerable evidence for ethnic disparities in maternal and pregnancy outcomes, with Black and South Asian women at increased risk of experiencing stillbirth, preterm birth, foetal growth restriction [3], maternal morbidity [2] and mortality [4].

Despite the widely accepted definition of reproductive health referring to “all matters relating to the reproductive system and to its functions and processes” [5] there has been a tendency in research to examine different aspects of reproductive health in isolation from one another. Commonly reported prevalence estimates of individual reproductive health conditions (e.g., “1 in 10 women suffer from endometriosis”) imply that such issues are only experienced by the (albeit substantial) minority. To estimate the proportion of people who experience any facet of poor reproductive health, detailed data collection focusing on the range of potential health issues experienced across the reproductive life course is required within a single sample.

In 2022, the UK Government launched the Women's Health Strategy for England, with reproductive health issues identified as priority areas [6]. As a commitment of this strategy, the Department of Health and Social Care (DHSC) commissioned the Women's Reproductive Health Survey for England 2023 (RHSE2023). This was the first population‐based survey carried out in Britain to cover such a wide range of questions relating to many aspects of reproductive health. Using this data, this paper aims to quantify the burden of poor reproductive health at the population‐level, broadly defined, by age, ethnicity, and financial security.

## 2 Methods

### 2.1 Recruitment

Based on the pilot study carried out in 2021 (see McCarthy et al. [7] for detail), we used online methods to recruit participants for the RHSE2023, resulting in a non‐probability convenience sample, with the aim of achieving a sample broadly reflective of the population in terms of age, ethnicity, education level, and region of residence. Participants were eligible to complete the survey if they were assigned female at birth, aged 16–55 years, and resident in England.

The sample achieved in the pilot study had an under‐representation of those from Black, Asian, and minority ethnic groups, those aged 24 and under, and those without a degree or equivalent education. Due to resource limits, we were unable to employ alternative sampling methodologies for this main survey. Therefore, we revised the recruitment materials and strategy with the aim of increasing recruitment among these groups. The recruitment strategy involved three strands: paid‐for advertising on social media (Instagram and Facebook); social media posting and dissemination to networks by partner organisations (LGBT Foundation; Race Equality Foundation; Brook; and Birth Companions); and press releases issued by DHSC and LSHTM at the survey launch to increase news coverage.

The survey advertisements and promotional materials featured newly developed illustrations (as opposed to the stock images used in the pilot survey) created by a graphic design company, and with input from our partner organisations, to reflect diversity in terms of age, ethnicity, and socio‐economic status. We used the Meta Ads Manager to create and deliver paid‐for adverts on Facebook and Instagram, and employed the same targeting strategies as in the pilot study [7], but implemented them from survey launch instead of waiting for the under‐representation of certain groups to be evident in the data collected, as in the pilot. For example, additional funds were directed to ensure more adverts were displayed to those living in local authority areas with a higher proportion of people from minority ethnic groups, to people aged 16–24, and to those who recorded lower levels of education on their social media profiles. All adverts featured one of the newly‐developed images alongside the same generic wording, (“Are you a woman aged 16‐55 years? Complete the Reproductive Health Survey for England 2023”; “Complete the Reproductive Health Survey for England 2023”). The word ‘woman’ was omitted from adverts designed to appeal to other gender identities who could be affected by the survey topics. Targeted language highlighting the groups of people at risk of under‐representation and emphasising the importance of their engagement was used in social media posts and outreach efforts delivered by our partner organisations.

The survey ran for 6 weeks, from 7th September to 19th October 2023. The sample was monitored once a week to assess how the digital marketing and communications strategy was working through comparison of the sample with Census 2021 data and to identify when additional efforts were needed to improve uptake among specific demographic groups.

### 2.2 Data Collection

On clicking the survey link, participants were taken to the information sheet, consent form, and self‐completion questionnaire hosted by Snap Surveys (https://www.snapsurveys.com/), an online survey platform. The survey was designed to be completed within 20 min, and filter questions ensured participants were only presented with questions of relevance to them.

Topics covered in the questionnaire included menstruation and menopause, family planning, pregnancy outcomes, reproductive morbidities, and experiences of care and support. The questionnaire was originally developed for the pilot survey based on the creation of a matrix of reproductive health stages and thematic concepts relating to the fulfilment of reproductive intentions, supporting reproductive wellness, and identification of reproductive morbidities. Existing survey instruments were mapped onto this matrix, and where gaps remained, new questions were designed and underwent cognitive testing [7]. Patient and Public Involvement Volunteers helped us to develop the questionnaire for the pilot study, ensuring that it was easy to understand and navigate, and covered topics relevant to people's lives. Further refinements were made to the questionnaire for the RHSE2023 based on learnings from the pilot study. Additional questions were added to the questionnaire based on our review of the topics covered in the recently published policy document, the Women's Health Strategy for England [6].

### 2.3 Measures

In this analysis, we report on 13 indicators of reproductive health organised into three domains: reproductive morbidities (diagnosed conditions which affect the reproductive organs of those assigned female at birth), poor menstrual health (potentially problematic symptoms directly related to the menstrual cycle), and pregnancy‐related adverse experiences (experiences that are relevant for fulfilment of reproductive intentions).

Within reproductive morbidities, we examined the proportion of participants who reported currently having: polycystic ovary syndrome (PCOS); endometriosis; uterine fibroids; uterine or cervical polyps; pelvic organ prolapse; cervical, ovarian, uterine, or breast cancer; or another reproductive health condition.

For poor menstrual health, we looked at the proportion of participants who reported having experienced heavy menstrual bleeding in the last year, severely painful periods in the last year, and for those aged 40 or over, the proportion reporting having experienced hot flushes and/or night sweats in the last year as potential peri‐menopausal or menopausal symptoms.

In relation to pregnancy‐related adverse experiences, we focused on the experience of pregnancy loss in the last year (including miscarriage, ectopic pregnancy, or stillbirth); infertility (as indicated by having sought NHS treatment for infertility in the last year); and whether participants had an unplanned pregnancy in the last year (measured using the London Measure of Unplanned Pregnancy, categorised as unplanned based on a score of 0–3) [8].

For details of the survey questions used for each indicator, see Table S1.

To ask about the participants' ethnicity, we used the same question and ethnic group categorisations as in the UK Census 2021 [9]. To capture financial security, we use a question designed to capture the participant's subjective financial situation, “How well would you say you yourself are managing financially these days?” with response options: ‘finding it very difficult’, ‘finding it quite difficult’, ‘just about getting by’, ‘doing alright’ and ‘living comfortably’ [10].

### 2.4 Analysis

We provide the demographic characteristics of the sample recruited, alongside the equivalent estimates from the 2021 Census. We present the percentages of participants who reported experiencing each indicator of reproductive health, followed by the percentage who reported at least one of these indicators within each domain and across all three combined. In order that our estimates could be used to indicate population‐level burden, the denominators of each percentage included all participants. For example, the question about heavy menstrual bleeding was only asked of those who reported having had a period in the last year, but our denominator also includes those who had not had a period in the last year.

Results are presented by five‐year age group and overall. Logistic regression analysis was used to examine differences in the burden of poor reproductive health by ethnic group and subjective financial situation while adjusting for age group. Due to the over‐representation of younger participants in our sample, particularly those aged 20–34 years, we applied weights based on the Census 2021 [9] age distribution for females. All analyses were carried out using Stata 18 [11].

## 3 Results

In the 6 weeks the survey was live, 59,332 eligible participants responded. A description of the socio‐demographics of the sample is provided in Table 1. In comparison to data from the 2021 Census, our sample had an over‐representation of younger age groups and those with higher levels of education, and an under‐representation of those from minority ethnic groups. However, the regional distribution of participants was broadly consistent with the 2021 Census.

**TABLE 1:** Description of RHSE2023 participants and England census data.

	RHSE 2023 *N* (%)	Census 2021
Age		
16 to 19	3995 (6.7%)	*8.4%*
20 to 24	9159 (15.4%)	*11.4%*
25 to 29	9931 (16.7%)	*12.7%*
30 to 34	9904 (16.7%)	*13.7%*
35 to 39	7954 (13.4%)	*13.1%*
40 to 44	6708 (11.3%)	*12.3%*
45 to 49	5147 (8.7%)	*12.3%*
50 to 55	6534 (11.0%)	*16.0%*
Ethnicity		
White	53 687 (92.3%)	*77.9%*
Mixed/multiple ethnic groups	1931 (3.3%)	*2.9%*
Asian/Asian British	1457 (2.5%)	*11.6%*
Black/Black British/Carib./African	795 (1.4%)	*5.1%*
Other ethnic group	311 (0.5%)	*2.6%*
Has degree or equivalent		
No	15 738 (27.4%)	*58.6%*
Yes	41 639 (72.6%)	*41.4%*
Government Region		
North East	2056 (3.9%)	*4.5%*
North West	6333 (12.1%)	*12.9%*
Yorkshire and Humber	5512 (10.5%)	*9.5%*
East Midlands	4313 (8.2%)	*8.4%*
West Midlands	4307 (8.2%)	*10.3%*
East	5562 (10.6%)	*10.9%*
London	9342 (17.8%)	*18.2%*
South East	8687 (16.6%)	*16.0%*
South West	6364 (12.1%)	*9.4%*
Gender^a^		
Woman/girl	56 822 (96.2%)	
Non‐binary	1026 (1.7%)	
Woman/girl/non‐binary	277 (0.5%)	
Think of myself in another way	293 (0.5%)	
Non‐binary/Trans	167 (0.3%)	
Man/boy/Trans	100 (0.2%)	
All other responses	397 (0.7%)	
Index Multiple Deprivation (IMD) 2019 quintiles		
1 (most deprived)	6751 (12.9%)	
2	10 664 (20.3%)	
3	11 592 (22.1%)	
4	11 710 (22.3%)	
5 (least deprived)	11 759 (22.4%)	

*Note:* Census 2021 estimates are based on census data from women, resident in England, aged‐16‐55 only.

^a^ Participants were asked "Which of the options describes how you think of yourself?" and could select all answer options that apply. We present the 6 most common responses, followed by all other responses together.

Tables 2a and 2b present the percentage of participants reporting each indicator of reproductive health by age group and overall. Polycystic ovary syndrome was the most commonly reported reproductive morbidity (10.5%) followed by endometriosis (8.8%). Uterine fibroids, uterine or cervical polyps, pelvic organ prolapse, and reproductive‐cancers were less commonly reported but increased with advancing age. Over a quarter of participants reported at least one reproductive morbidity (28.0%); this was 12.9% among the 16–19‐year‐olds and ranged from 29.2% to 33.9% among those aged 30 and over.

**TABLE 2a:** Reproductive morbidities by age group and overall.

	16 to 19	20 to 24	25 to 29	30 to 34	35 to 39	40 to 44	45 to 49	50 to 55	Total
*Reproductive morbidities*									
PCOS	5.93	10.95	14.74	13.89	12.72	10.98	8.50	5.33	10.47
	[5.18, 6.79]	[10.28, 11.65]	[14.01, 15.5]	[13.18, 14.64]	[11.96, 13.53]	[10.2, 11.82]	[7.71, 9.35]	[4.77, 5.94]	[10.21, 10.74]
	3337	7921	8692	8595	6901	5781	4473	5652	51 352
Endometriosis	5.36	7.59	9.87	9.95	10.55	10.36	9.41	6.30	8.77
	[4.65, 6.18]	[7.02, 8.19]	[9.26, 10.52]	[9.33, 10.6]	[9.85, 11.3]	[9.60, 11.17]	[8.59, 10.3]	[5.69, 6.96]	[8.53, 9.03]
	3337	7921	8692	8595	6901	5781	4473	5652	51 352
Uterine fibroids	0.21	0.45	1.07	2.65	4.71	7.54	9.03	11.61	5.09
	[0.10, 0.44]	[0.33, 0.63]	[0.87, 1.31]	[2.33, 3.01]	[4.23, 5.24]	[6.89, 8.25]	[8.23, 9.91]	[10.80, 12.47]	[4.88, 5.30]
	3337	7921	8692	8595	6901	5781	4473	5652	51 352
Uterine or cervical polyps	0.06	0.42	0.77	1.08	1.42	2.40	2.35	3.31	1.60
	[0.02, 0.24]	[0.30, 0.59]	[0.61, 0.98]	[0.88, 1.32]	[1.17, 1.73]	[2.04, 2.83]	[1.94, 2.84]	[2.87, 3.81]	[1.49, 1.73]
	3337	7921	8692	8595	6901	5781	4473	5652	51 352
Pelvic organ prolapse	0	0.14	0.32	1.55	3.52	4.20	4.70	4.23	2.50
	—	[0.08, 0.25]	[0.22, 0.47]	[1.31, 1.83]	[3.11, 3.98]	[3.72, 4.75]	[4.11, 5.36]	[3.73, 4.79]	[2.36, 2.66]
	3337	7921	8692	8595	6901	5781	4473	5652	51 352
Cervical, ovarian, uterine, or breast cancer	0.06	0.03	0.14	0.12	0.39	0.57	1.01	1.47	0.52
	[0.02, 0.24]	[0.01, 0.10]	[0.08, 0.24]	[0.06, 0.22]	[0.27, 0.57]	[0.41, 0.80]	[0.75, 1.35]	[1.19, 1.82]	[0.46, 0.60]
	3337	7921	8692	8595	6901	5781	4473	5652	51 352
Other reproductive health condition	2.91	4.53	6.21	6.91	8.17	8.04	6.04	5.45	6.19
	[2.39, 3.53]	[4.10, 5.01]	[5.72, 6.74]	[6.39, 7.47]	[7.55, 8.84]	[7.37, 8.77]	[5.38, 6.77]	[4.89, 6.07]	[5.98, 6.40]
	3337	7921	8692	8595	6901	5781	4473	5652	51 352
Any of the above	12.86	20.57	27.23	29.31	33.01	33.85	31.59	29.18	28.0
	[11.76, 14.03]	[19.69, 21.47]	[26.31, 28.18]	[28.35, 30.28]	[31.91, 34.13]	[32.64, 35.08]	[30.24, 32.97]	[28.0, 30.37]	[27.6, 28.41]
	3337	7921	8692	8595	6901	5781	4473	5652	51 352

*Note:* Table presents: % [95% confidence interval] N. In order that our estimates can be used to indicate population‐level burden, the denominators of each percentage included all participants.

**TABLE 2b:** Menstrual health, and adverse‐pregnancy related experiences, by age group and overall.

	16 to 19	20 to 24	25 to 29	30 to 34	35 to 39	40 to 44	45 to 49	50 to 55	Total
Menstrual Health									
Severe period pain^a^	36.49	31.96	25.51	18.93	15.98	14.12	11.33	5.49	18.65
	[34.96, 38.04]	[31.0, 32.95]	[24.65, 26.4]	[18.16, 19.73]	[15.17, 16.81]	[13.29, 15.0]	[10.47, 12.24]	[4.95, 6.08]	[18.33, 18.97]
	3774	8763	9572	9533	7674	6429	4962	6268	56 975
Heavy menstrual bleeding^a^	62.44	53.58	42.68	37.48	37.93	40.96	37.34	21.97	40.04
	[60.89, 63.98]	[52.53, 54.62]	[41.69, 43.68]	[36.51, 38.46]	[36.85, 39.02]	[39.76, 42.16]	[36.0, 38.7]	[20.96, 23.01]	[39.62, 40.45]
	3773	8754	9564	9525	7673	6424	4952	6267	56 932
Hot flushes or night sweats^b^						52.5	67.97	76.15	66.57
						[51.26, 53.74]	[66.64, 69.27]	[75.07, 77.20]	[65.86, 67.27]
						6215	4824	6118	17 157
Any of the above	68.74	59.84	49.17	42.5	41.77	71.74	80.86	81.49	61.86
	[67.24, 70.2]	[58.81, 60.86]	[48.17, 50.17]	[41.51, 43.49]	[40.67, 42.88]	[70.61, 72.84]	[79.73, 81.94]	[80.5, 82.43]	[61.45, 62.26]
	3775	8757	9563	9523	7665	6302	4885	6179	56 649
Pregnancy‐related adverse experiences									
Unplanned pregnancy^c^	2.31	2.54	1.52	0.94	0.60	0.39	0.11	0.03	0.95
	[1.86, 2.86]	[2.22, 2.91]	[1.28, 1.79]	[0.76, 1.16]	[0.45, 0.81]	[0.26, 0.58]	[0.05, 0.26]	[0.01, 0.14]	[0.88, 1.03]
	3510	8217	8963	8856	7159	5958	4624	5837	53 124
Pregnancy loss in last year^c^	1.48	1.60	2.68	5.69	5.66	2.78	0.45	0.09	2.59
	[1.13, 1.93]	[1.35, 1.90]	[2.37, 3.04]	[5.23, 6.19]	[5.15, 6.22]	[2.40, 3.23]	[0.29, 0.69]	[0.04, 0.20]	[2.47, 2.72]
	3523	8247	9020	8975	7262	6034	4669	5904	53 634
Sought NHS fertility treatment in last year^d^	0.06	0.59	2.74	6.61	7.01	3.05	0.20	0.05	2.67
	[0.014, 0.28]	[0.44, 0.78]	[2.42, 3.10]	[6.11, 7.14]	[6.44, 7.63]	[2.64, 3.52]	[0.10, 0.38]	[0.02, 0.16]	[2.54, 2.80]
	3477	8141	8884	8795	7114	5936	4591	5761	52 699
Any of the above	2.83	4.03	6.11	12.06	12.0	5.53	0.66	0.14	5.52
	[2.33, 3.44]	[3.62, 4.48]	[5.63, 6.63]	[11.39, 12.76]	[11.26, 12.77]	[4.98, 6.15]	[0.46, 0.94]	[0.07, 0.28]	[5.33, 5.71]
	3461	8122	8865	8765	7077	5874	4538	5685	52 387
Any reproductive, menstrual, or pregnancy issue									
Any reproductive, menstrual, or pregnancy issue	74.19	69.07	63.33	61.48	63.14	81.83	86.79	87.3	73.72
	[72.75, 75.59]	[68.07, 70.05]	[62.34, 64.32]	[60.47, 62.49]	[62.02, 64.25]	[80.85, 82.78]	[85.81, 87.72]	[86.44, 88.12]	[73.34, 74.09]
	3631	8402	9071	8965	7171	6148	4801	6073	54 262

*Note:* Table presents: % [95% confidence interval] N. In order that our estimates can be used to indicate population‐level burden, the denominators of each percentage included all participants. Denominators vary due to item non‐response.

^a^ Denominator includes all participants regardless of whether they have had a period in the last year.

^b^ Denominator includes all participants aged 40 to 55 years.

^c^ Denominator includes all participants regardless of whether they have had a pregnancy in the last year.

^d^ Denominator includes all participants regardless of whether they have ever experienced infertility.

Overall, 18.7% of participants reported experiencing severe period pain in the last year, but this varied considerably by age, peaking among 16–19‐year‐olds and 20–24‐year‐olds at 36.5% and 32%, respectively, and falling to 5.5% among 50–55‐year‐olds. Heavy menstrual bleeding was reported by 40% of the sample and also peaked at younger ages (62.4% of 16–19 year olds and 53.6% of 20–24 year olds). Among those aged 40 or over, two‐thirds reported experiencing hot flushes and/or night sweats. Looking across these indicators of menstrual health, 61.9% of participants had experienced at least one issue in the last year, with the highest menstrual health burden among the youngest (68.7%) and oldest (81.5%) age groups.

In relation to our indicators of pregnancy‐related adverse experiences, just under 1% of the sample had an unplanned pregnancy in the last year, with the highest among those aged 20–24 years, at 2.5%. Pregnancy loss was most commonly reported by participants in their thirties (5.7%), and the proportion seeking NHS fertility treatment followed a similar pattern, peaking at around 7% in this age group. Overall, 1 in 20 participants (5.5%) reported at least one of these pregnancy‐related adverse experiences in the last year, and this proportion reached 12% among those aged 30–39.

Looking across all three domains, the overall proportion reporting any reproductive, menstrual, or pregnancy‐related issue was 73.7%, ranging from 61.5% among 30–34‐year‐olds to 87.3% among participants aged 50–55‐years.

Table 3 presents the proportion of participants reporting any reproductive morbidity and any pregnancy‐related adverse experience by ethnic group and by financial situation, and the corresponding age‐adjusted odds ratios. Within the menstrual health domain, the indicators of heavy and/or severely painful periods are presented separately to the menopausal symptoms. Compared with White participants, those from minority ethnic groups had significantly greater odds of reporting a reproductive morbidity, peaking among Black, Black British, Caribbean or African participants (aOR: 1.69, 95% CI: 1.43–1.98). Further analyses found this to be driven by a large difference in the percentage reporting uterine fibroids: 19.8% (95% CI: 16.7–23.2) among Black ethnic groups and 9.8% (95% CI: 6.4, 14.7) among other ethnic groups, compared with 4.9% (95% CI: 4.7–5.1) among White ethnic groups. Participants from minority ethnic groups were also significantly more likely to report heavy and/or severely painful periods, while little variation was observed in relation to hot flushes and/or night sweats. Finally, pregnancy‐related adverse experiences were most commonly reported by Black, Black British, Caribbean or African participants (aOR: 1.50, 95% CI: 1.14–1.99) and those from other ethnic groups (aOR: 1.91, 95% CI: 1.25–2.90). The odds of reporting any reproductive morbidity, heavy and/or severely painful periods, and menopausal symptoms, increased with declining financial security. While no statistically significant pattern was observed for the adverse pregnancy experiences domain overall, further analyses indicated a strong association between increasing financial insecurity and odds of having had an unplanned pregnancy in the last year; those who reported that they were ‘finding it very difficult’ had almost five‐times greater odds of an unplanned pregnancy in the last year compared to those who were ‘living comfortably’ (aOR: 4.96, 95% CI: 3.23–7.41).

**TABLE 3:** Age‐adjusted associations between ethnicity, financial situation, and reproductive health.

	Any reproductive morbidity			Heavy and/or severely painful periods			Hot flushes and/or night sweats			Any pregnancy‐related adverse experience		
	%	aOR (95% CI)	*N*	%	aOR (95% CI)	*N*	%	aOR (95% CI)	*N*	%	aOR (95% CI)	*N*
Ethnic group												
White	27.7	1 (ref)	47 205	43.6	1 (ref)	52 251	66.7	1 (ref)	16 136	5.4	1 (ref)	48 196
Mixed or multiple ethnic groups	28.8	1.14*	1698	50.8	1.14**	1874	67.6	1.15	352	6.2	1.04	1733
		(1.02, 1.27)			(1.03, 1.26)			(0.91, 1.45)			(0.85, 1.27)	
Asian or Asian British	31.6	1.27***	1230	49.1	1.12*	1406	60.2	0.82	332	6.7	1.15	1235
		(1.12, 1.44)			(1.00, 1.25)			(0.65, 1.03)			(0.92, 1.43)	
Black, Black British, Caribbean or African	38. 1	1.69*	650	52.7	1.28**	749	64.1	0.97	145	9.0	1.50**	655
		(1.43, 1.98)			(1.10, 1.50)			(0.68, 1.38)			(1.14, 1.99)	
Other ethnic group	34.2	1.36*	259	50.5	1.36*	295	62.2	0.79	82	10.5	1.91**	257
		(1.04, 1.78)			(1.06, 1.75)			(0.50, 1.25)			(1.25, 2.90)	
Financial situation												
Living comfortably	26.0	1 (ref)	10 924	34.9	1 (ref)	12 202	63.3	1 (ref)	4800	5.7	1 (ref)	11 191
Doing alright	27.2	1.13*	22 752	42.4	1.23***	25 245	66.0	1.17***	7526	5.4	0.91	23 223
		(1.07, 1.20)			(1.18, 1.29)			(1.08, 1.27)			(0.83, 1.00)	
Just getting by	29.9	1.38*	1068	50.5	1.60***	13 350	70.1	1.46***	3414	5.5	0.96	12 285
		(1.30, 1.47)			(1.52, 1.69)			(1.32, 1.60)			(0.86, 1.07)	
Finding it quite difficult	30.0	1.46***	4076	56.3	1.91**	4460	71.7	1.58***	945	5.7	1.03	4148
		(1.34, 1.59)			(1.77, 2.05)			(1.35, 1.86)			(0.88, 1.20)	
Finding it very difficult	36.1	1.87***	1396	60.1	2.39***	1508	74.0	1.77***	401	6.2	1.18	1408
		(1.65, 2.12)			(2.13, 2.68)			(1.40, 2.24)			(0.94, 1.49)	

*p ≤ 0.05.

**p ≤ 0.01.

***p ≤ 0.001.

## 4 Discussion

### 4.1 Main Findings

This is the first study to simultaneously examine this combination of indicators of reproductive health within a single sample. Our findings indicate there is a substantial burden of poor reproductive health and that the nature of this burden varies by age group. Reproductive morbidities tended to increase with advancing age, while menstrual‐related issues followed a u‐shaped curve peaking at both younger and older ages, with pregnancy‐related adverse experiences most commonly experienced between the ages of 30 and 39 years. We found evidence of inequalities in reproductive health according to ethnicity and financial security. Minority ethnic groups, and particularly those identifying as Black, Black British, Caribbean, or African, experienced a higher burden of poor reproductive health across the three domains investigated, while greater financial insecurity was associated with poor reproductive health across two domains.

### 4.2 Strengths and Limitations

The main strength of this study is the collection of data relating to a wide range of indicators relevant to reproductive health within a single large population‐based sample. Several limitations should also be noted in the interpretation of our findings. Having used a non‐probability convenience approach to sampling, we cannot be certain of the extent to which our findings are representative of the wider population of women and those assigned female at birth aged 16–55 living in England. In comparing the characteristics of the RHSE participants to UK census data, our sample has an under‐representation of Black, Asian, and other minority ethnic groups and those without degree‐level education. Therefore, the sample achieved is likely to under‐represent those at greater risk of having poorer reproductive health and outcomes. Comparing our individual estimates for each of the reproductive conditions/experiences to published literature (see next section) provides reassurance against concerns of over‐estimating the burden of poor reproductive health. Due to resource constraints, the sample was limited to those aged 16 to 55 years; however, our findings provide clear evidence that reproductive health issues show no sign of declining with advancing age, meaning an important segment of the population is missed in this analysis. Furthermore, although broad, the measures included in our study fail to include important experiences relevant to reproductive health; for example, we do not include indicators relating to recent birth‐related issues such as psychological trauma and obstetric injury, ante‐ and post‐natal poor mental health, menstrual‐related mood disorders, other gynaecological symptoms such as vulval pain and non‐menstrual pelvic pain, and broader symptoms of peri‐menopause and menopause, such as brain fog. We also do not report the psychological, relational, and economic impacts of the reproductive health experiences considered, thereby capturing only their occurrence, but not their broader costs. While our analyses focused on age, ethnicity, and financial security, other factors are likely to relate to reproductive health status, such as access to care and general health status. For those conditions included, we necessarily rely on participants' self‐report, but it is possible that certain experiences and conditions may be over or under‐reported according to their recent salience, the time taken to diagnosis, and whether treatment or strategies to control symptoms have been effective. As a proxy for recent experience of infertility, we relied on a question asking participants whether they had tried to access free fertility treatment on the NHS in the last year, which will not capture those who proceeded directly to private providers. However, given that private treatment is only accessible to those who can afford it, its inclusion as an indicator may have biased our results, particularly when examining experiences by financial status. Finally, while useful for examining burden at a population level, we have combined clinically‐distinct conditions and experiences into three domains. The inequalities observed for each domain according to ethnicity and financial status may not be reflected for each individual indicator separately.

### 4.3 Interpretation

Due to our sampling strategy, we cannot claim the sample is necessarily representative of the wider population, and the comparison of our data with the 2021 Census shows that particular sociodemographic groups were under‐represented in the sample achieved. In considering the impact that our sample make‐up may have on the estimates reported, those who are under‐represented (minority ethnic groups, lower education, living in more deprived areas) are generally the same groups who are likely to have poorer reproductive health and outcomes. On this basis, our findings may be an under‐estimate of the true burden of poor reproductive health in the wider population. However, we would argue that having an indicative lower bound is more useful than a potential over‐estimate. Additionally, those over‐represented in our sample may be more likely than the general population to access healthcare services and receive a diagnosis of certain gynaecological conditions, which can take many years and visits before adequate investigations are carried out. In comparing our estimated prevalences of each individual indicator of reproductive health to previously published figures, we do not find evidence to suggest an over‐representation of any aspect of poor reproductive health. Our estimates for PCOS and endometriosis were consistent with previously reported prevalence rates [12, 13], however, those for fibroids, polyps, and prolapse were considerably lower than those reported in other studies, potentially due to the under‐diagnosis of these conditions [14, 15, 16].

Published estimates of heavy menstrual bleeding and pain vary widely due to differences in measurement and sampling approaches, making comparisons difficult; however, a survey of European respondents found 27% reported at least two symptoms of heavy menstrual bleeding [17]; while a cross‐sectional study across 10 low‐and middle‐income countries reported prevalences of heavy menstrual bleeding ranging from 38.3% to 77.6%, as measured by the multi‐item SAMANTA scale [18]. In a postal survey conducted in Scotland, 35% of participants reported ‘heavy’ or ‘very heavy’ periods, and 15% reported ‘severe’ or ‘very severe’ period pain [19]. In relation to menopausal symptoms, another online survey among 35 to 70‐year‐olds living in the UK found that 80.7% of participants reported recently experiencing hot flushes or night sweats [20].

Our estimate of unplanned pregnancy in the last year is slightly lower than the 1.5% among 16–44 years old in the third National Survey of Sexual Attitudes and Lifestyles [21], even when restrictions are made to calculate this among the same age range. In relation to pregnancy loss, it is estimated that around 15% of all recognised pregnancies end in miscarriage [22], though the true rate is likely to be higher. Our results show a peak in the proportion of women reporting pregnancy loss in the last year among those aged 30 to 39, as would be expected given that conception rates are highest among the 30–34‐year‐old age group in England and Wales [23], and the risk of miscarriage increases with maternal age [24]. In line with these findings, the proportion of participants reporting having sought fertility treatment from the NHS in the last year follows a similar pattern across the age groups, reflective of when people may be most likely to attempt to have children and the increased risk of infertility that comes with advancing age.

Our findings of disparities in reproductive health according to ethnicity and financial status are consistent with the large body of literature concerned with socio‐economic inequalities in health. Driving the ethnic inequality observed for the reproductive morbidity domain was a stark difference in the proportion of Black respondents reporting uterine fibroids compared to other ethnicities. This pattern has been repeatedly demonstrated in US‐based studies with a two‐to three‐fold increase in the risk of uterine fibroids among Black women compared to White women [25, 26, 27], though the relative contribution of social, environmental, and biological factors is not well understood [28, 29, 30]. In interpreting the association between self‐rated financial security and reproductive health, various mechanisms should be considered; for example, those with greater financial insecurity may face barriers in accessing effective care for the management of menstrual symptoms; poor reproductive health itself may be disruptive to a person's ability to work and maintain financial security; or the potential impact of poor reproductive health on health more generally may in turn contribute to the way in which one interprets their own financial security (e.g., prior research using the same measure of financial status found evidence for causal effects of general and mental health status on self‐rated financial security) [31].

### 4.4 Implications

Our findings indicate that reproductive health issues are experienced by the majority of women and those assigned female at birth. While increased risks of poor maternal health among Black women and other minority ethnic groups are well documented [32], this analysis demonstrates that ethnic inequalities are evident across multiple aspects of reproductive health. Furthermore, previous research has often relied on area‐level indicators of deprivation as a proxy for individual‐level disadvantage in examining socio‐economic disparities in reproductive health; our findings show that inequalities exist according to person‐level self‐rated financial security. These findings are set in a context where women's experiences of health and reproductive conditions are often ignored, dismissed or simply considered within the norm of what one should expect [33]. Funding and meaningful action to promote and support women's health has historically being lacking, and according to the UK Clinical Research Collaboration, just 2.4% of health and biomedical research funding was spent on ‘Reproductive Health and Childbirth’ in 2022 [34]. Women's health services are under increasing pressure, with an estimated 591 000 people in England currently on a waiting list for gynaecology hospital care [35], and a series of public inquiries highlighting serious failings in the provision of maternity services [36]. Further implications of our findings for policy and future research are presented in Box 1.

BOX 1 **Implications for policy and future research.**

- Given the sampling strategy employed, we cannot claim to have produced perfect prevalence estimates, and it is likely that our results provide an indicative lower bound of the population‐level burden of poor reproductive health. This extent of poor reproductive health may not seem surprising to those providing specialised clinical care. However, our data provide the first population‐based estimates for a wide range of reproductive health issues simultaneously, which can inform investment, commissioning, and policy‐making, and increase awareness among non‐specialist healthcare providers.
- Reproductive health is underfunded and under‐researched. A recent report by the UK House of Commons Women and Equalities Committee highlighted the neglect of reproductive health in policy, the provision of healthcare, and in medical research [37]. Our findings highlight that there is no empirical basis for reproductive health to be treated as niche and unimportant.
- With the change of UK government last year, the policy steer for women's reproductive health in England is not yet clear. The Women's Health Strategy for England of the former government was backed up with inadequate levels of funding for Integrated Care Boards to establish and sustainably expand the proposed ‘Women's Health Hubs’, despite economic analyses indicating that for every £1 spent on implementing a Primary Care Network‐sized hub, there would be an estimated £5 of benefits in return [36, 38]. A grasp of reproductive health indicators at a population level are important to prioritise this key area of health.
- Poor reproductive health impacts other aspects of health and general wellbeing, and threatens the extent to which those affected are able to engage in education and work, meaning the costs extend beyond the individual to society.
- Primary care will most often be the first port of call for reproductive healthcare. It is important that those working in primary care appreciate how common reproductive health issues are, as well as how their distribution varies according to age, so that referrals to the appropriate specialised services are not delayed unnecessarily.
- Our findings highlight the need for greater investment in the conduct of high‐quality research focused on reproductive health in the population, ensuring adequate representation from those groups at risk of poorer health and outcomes and who may be better reached by more resource‐intensive offline community‐based methods of recruitment and the opportunity to contribute to such research in languages other than English.
- In addition to public health research, our findings also underscore the need for genuine investment in clinical medical research so that the huge number of people affected by poor reproductive health can benefit from improved diagnostics and new treatment options.

Recently there has been increasing interest in supportive workplace policies for specific issues such as pregnancy loss [39] and menopause [40], though these only relate to two reproductive health issues and fail to recognise that the most salient and disruptive reproductive experiences will differ according to age and life stage. Our findings reinforce the value of taking a life‐course approach to reproductive health and the importance of support and healthcare being delivered as a continuum [41].

## 5 Conclusion

Examining multiple indicators simultaneously has exposed a substantial burden of poor reproductive health experienced by women and those assigned female at birth, the nature of which varies considerably by age. Ethnic and financial inequalities exist across multiple facets of reproductive health. Investment and innovation in health services and support strategies are urgently needed to mitigate the impact of poor reproductive health on the lives of women and those assigned female at birth.

## Author Contributions

R.S.F., M.J.P., and O.L.M. designed the study, including questionnaire development, recruitment strategy, and data collection. M.J.P. carried out the data analysis for this article, with input from O.M. and R.S.F., M.J.P., wrote the first draft of the article, and all authors were responsible for reviewing and editing it. All authors have read and approved the final version for publication.

## Ethics Statement

Approval for this study was granted by the London School of Hygiene and Tropical Medicine Ethics Committee (LSHTM Ethics Ref: 29389) on 25th July 2023.

## Conflicts of Interest

The authors declare no conflicts of interest.

## Supporting information


Table S1.

## Data Availability

The data that support the findings of this study are available from Department of Health and Social Care. Restrictions apply to the availability of these data, which were used under license for this study. Data are available from the author(s) with the permission of Department of Health and Social Care.
