# Supplementary material for: The Burden of Poor Reproductive Health in England: Results From a Cross‐Sectional Survey
Source: BJOG. 2025 Mar 24;132(13):2052–63. doi: 10.1111/1471-0528.18133 (PMC12592767; doi:10.1111/1471-0528.18133)
Supplement: Supplementary file 1 — Table S1. [file BJO-132-2052-s001.docx]

***Supporting Information***

***Table S1: Survey questions for variables used in analysis***

| **Domain** | **Survey question(s)** | **Derivation of variable** |
| --- | --- | --- |
| **Reproductive morbidities** | *Do you currently have any of the following conditions? Tick all that apply*    *Polycystic ovary syndrome* (*PCOS); endometriosis; uterine fibroids; uterine or cervical polyps; pelvic organ prolapse; cervical, ovarian, uterine, or breast cancer; other reproductive health condition (please specify); don’t know; none of the above*. | Seven binary variables were derived for each reproductive morbidity. |
| **Poor menstrual health** | Question asked to those who reported having had a period in the last year:  *In the last year, in general, have you had heavy bleeding during your period (for example, where you have had to change your pad, tampon or menstrual cup every hour or two)?*  *Yes*  *No*  *Unsure* | Those who answered ‘yes’ were coded as having experienced heavy menstrual bleeding. |
|  | Question asked to those who reported having had a period in the last year:  *In the last year, in general, how much pain do you experience during your periods? (this may feel like a sharp stabbing sensation, a dull ache, cramps or feelings of 'heat' coming out of your tummy)*    *No pain*  *Mild pain*  *Moderate pain*  *Severe pain* | Those who answered ‘severe pain’ were coded as having experience severe menstrual pain. |
|  | Question asked to those aged 40 or above:  *In the last year, have you experienced the following symptoms, which may be related to peri-menopause or menopause? Tick all that apply*  *Hot flushes*  *Night sweats*  *Vaginal dryness*  *Discomfort during sex*  *Reduced sex drive (libido)*  *Difficulty sleeping*  *Low mood or anxiety*  *Problems with memory or concentration*  *None of these* | Those who answered ‘hot flushes’ or ‘night sweats’ were coded as having experienced these menopausal symptoms. |
| **Pregnancy-related adverse experiences** | Questions asked to those who reported every having been pregnant:  *In the last year, have you had a miscarriage (loss of a pregnancy before 24 weeks)?*  *Yes*  *No*  *Have you ever had an ectopic or tubal pregnancy (a pregnancy where a fertilised egg implants itself outside of the uterus, usually in the fallopian tubes)?*  *Yes*  *No*  *How long ago was your ectopic pregnancy?*  *Within the last 12 months*  *1 year ago*  *2 years ago*  *3 years ago*  *4 years ago*  *Five or more years ago*  *Have you ever had a stillbirth (death of a baby at or after 24 weeks of pregnancy)?*  *Yes*  *No*  *How long ago was your stillbirth?*  *Within the last 12 months*  *1 year ago*  *2 years ago*  *3 years ago*  *4 years ago*  *Five or more years ago* | Those who reported having had a miscarriage or ectopic pregnancy or stillbirth in the last year were coded as having experienced pregnancy loss. |
|  | *In the last year, have you tried to access free fertility treatment on the NHS?*  *Yes*  *No* | Those who answered yes were coded as having sought treatment for infertility. |
|  | *Because you said you experienced a pregnancy in the last year, we'd like to ask you more questions about your last pregnancy*  *In the month that I became pregnant...*  *I /we were not using contraception*  *I/we were using contraception, but not on every occasion*  *I/we always used contraception, but knew that the method, had failed (i.e. broke, moved,*  *came off, came out, not worked etc.) at least once*  *I/we always used contraception*  *In terms of becoming a parent (first time or again), I feel*  *that my pregnancy happened at the...*  *right time*  *ok, but not quite right time*  *wrong time*  *Just before I became pregnant...*  *I intended to get pregnant*  *my intentions kept changing*  *I did not intend to get pregnant*  *Just before I became pregnant...*  *I wanted to have a baby*  *I had mixed feelings about having a baby*  *I did not want to have a baby*  *The next question asks about your partner. This might be (or have been) your husband/wife/civil partner, a partner you live with, a partner who lives elsewhere, someone you have had sex with once or twice, or a parenting (non-romantic) partner.*  *Before I became pregnant....*  *My partner and I had agreed that we would like me to be pregnant*  *My partner and I had discussed having children together, but had not agreed for me to get*  *pregnant*  *We never discussed having children together*  *I chose to become pregnant without a partner*  *Before you became pregnant, did you do anything to improve your health in preparation for pregnancy?*  *Tick all that apply*  *took folic acid*  *stopped or cut down smoking*  *stopped or cut down drinking alcohol*  *ate more healthily*  *sought medical/health advice*  *took some other action*  *I did not do any of the above before my pregnancy* | These questions form the London Measure of Unplanned Pregnancy. Those scoring 0-3 on this measure were coded as having had a unplanned pregnancy. See: <https://lmup.org.uk/>. |
